# The Degeneration and Apoptosis Patterns of Cone Photoreceptors in* rd11* Mice

**DOI:** 10.1155/2017/9721362

**Published:** 2017-01-12

**Authors:** Hua Zhang, Xia Li, Xufeng Dai, Juanjuan Han, Yangyang Zhang, Yan Qi, Ying He, Yan Liu, Bo Chang, Ji-jing Pang

**Affiliations:** ^1^School of Ophthalmology & Optometry, The Eye Hospital, Wenzhou Medical University, Wenzhou, Zhejiang 325027, China; ^2^Department of Ophthalmology, First Affiliated Hospital of Guangxi Medical University, Nanning, Guangxi 530021, China; ^3^Fenyang College, Shanxi Medical University, Fenyang, Shanxi 032200, China; ^4^Qingdao Eye Hospital, Shandong Eye Institute, Shandong Academy of Medical Science, Qingdao 266000, China; ^5^The Jackson Laboratory, Bar Harbor, ME, USA; ^6^Department of Ophthalmology, University of Florida, Gainesville, FL, USA

## Abstract

The retinal degeneration 11 (*rd11*) mouse is a new animal model with rapid photoreceptor degeneration. The long-term efficacy of gene therapy has a direct relationship with the onset of photoreceptor degeneration or apoptosis, whereas the degeneration or apoptosis patterns of photoreceptors are still unclear in* rd11* mice. The distribution patterns of cone function-related L- and S-opsin were examined by immunofluorescence staining, and the apoptosis was performed by TUNEL assay in* rd11* mice. The expression pattern of L-opsin or S-opsin in* rd11* retina at postnatal day (P) 14 was similar to the pattern observed in wildtype retina. With increasing age, the expression of L-opsin and S-opsin, especially S-opsin, decreased significantly in* rd11* mice. The degeneration of L-opsin began around the optic nerve and expanded to the periphery of the retina, from the ventral/nasal to dorsal/temporal retina, whereas the expression of S-opsin gradually decreased from the dorsal/temporal to ventral/nasal retina. Apoptotic signal appeared at P14 and was strongest at P28 of* rd11* mice. The key genes associated with apoptosis confirmed those changes. These indicated that the degeneration and apoptosis of cone photoreceptors began at P14 of* rd11* mice, which was a key point for gene therapy.

## 1. Introduction

Retinal degenerative diseases such as retinitis pigmentosa and Leber congenital amaurosis (LCA) are one of the most common causes of visual impairment and blindness. At present, there are no good treatment methods for retinal degenerative diseases, especially the inherited retinal degenerative diseases, but gene replacement therapy may be a breakthrough. Adeno-associated virus (AAV) vector by subretinal injection could transfect retinal pigment epithelium and photoreceptor cells, which has been confirmed by clinical trials of gene therapy from human LCA2 and choroideremia [1, 2]. In recent years, gene therapy in animal models has made a rapid development [3–6].

Since retinal degeneration 1 (*rd1*) mice were first used to study the inherited retinal degenerative diseases, many additional mouse models with retinal degeneration were found and are widely used [7–9]. Recently, a new retinal degeneration model,* rd11* mice, was identified, which exhibits rapid photoreceptor degeneration followed by the loss of rod and then cone cells and could better simulate the human inherited retinal degenerative diseases [5, 10]. Natural mutation of the gene lysophosphatidylcholine acyltransferase 1 (*Lpcat1*) leads to rapid decline of rod photoreceptors function and the subsequent decline of cone photoreceptors function in the early stages of* rd11* mice development.* Lpcat1* encodes a phospholipid synthesis and remodeling enzyme [11], which plays an important role in metabolism in the early photoreceptor cell membrane.

Opsin is a major component of the light-sensitive material and is the first leg of eye sensitivity to light. The gene mutation and structural abnormality of opsin can cause the visual impairment [12–14]. Rodent retinas have two types of photoreceptors, rods (97% of total photoreceptors) and cones (3% of total photoreceptors), and opsins are divided into rod-opsin and cone-opsin, which are expressed in the rod photoreceptors and cone photoreceptors, respectively [15]. According to the range of absorption spectra, the cone-opsins can be further divided into two different subgroups, middle-to-long-wavelength opsin (L-opsin, also referred as M-opsin in some references) and short wavelength opsin (S-opsin), in the vision system of mice. L-opsin is sensitive to red and green light, whereas S-opsin is sensitive to ultraviolet light [16, 17]. In earlier studies, L-opsin was found to be mainly expressed in the dorsal retina and S-opsin in the ventral retina [18]; however, recent studies have shown that L-opsin and S-opsin can be coexpressed in most common cone photoreceptors throughout the retina [19–22]. The distribution of L-opsin decreased from the dorsal to ventral position, whereas S-opsin was relatively consistently distributed [19]. L-opsin was expressed in all cone photoreceptors, but S-opsin was repressed in the far dorsal retina [21].

It has been reported that gene replacement therapy by subretinal injection can restore the vision function in animal and human eyes with retinal degenerative diseases [1, 5, 23]. However, the recent long-term clinical trial data related to middle to late stages of LCA2 eyes showed that the degeneration processes of remaining photoreceptor cells did not completely stop in treated areas [24]. In LCA dog models, the treatment before the onset of retinal degeneration could significantly improve the visual function and prevent the loss of photoreceptor cells; but loss of photoreceptor cells continues slowly after the onset of degeneration [24]. So the loss of photoreceptor cells after gene therapy had a direct relationship with the onset of retinal degeneration at the time of treatment, and determining the start time of degeneration is critical for gene therapy.

The study of the distribution of cone-opsins throughout retina is important for our understanding of photoreceptor degeneration. In separate studies, Gargini et al. and Li et al. analyzed the characteristics of cone cells degeneration using immunocytochemical analysis of whole mount retina sections, which are widely used to identify retinal photoreceptor cells, bipolar cells, horizontal cells, amacrine cells, Müller cells, and retinal pigment epithelial cells (RPE) in mice and other mammals [23, 25, 26]. Because the cone-opsins play a major role in the process of cone photoreceptor degeneration [27], research on cone-opsin is one of the hotspots in the field of photoreceptor degeneration.

The photoreceptor degeneration and apoptosis are inseparable [28]. Caspase cascade is the core of apoptosis [29]. The antiapoptotic factor B-cell lymphoma-2 (Bcl-2) and Bcl-2-associated death promoter (Bad) can inhibit caspase cascade, whereas proapoptotic factor BH3 interacting domain death agonist (Bid) and Bcl-2-associated X (Bax) can activate caspase cascade [30]. Some factors can regulate the expression of Bad, Bid, and Bax in the process of photoreceptor degeneration [28, 30].

In this article, we analyze the changes in cone-opsin expression during photoreceptor degeneration using immunocytochemical analysis with retinal whole mount and frozen sections and apoptosis process by TUNEL staining in* rd11* mice. It is very important for gene therapy to clarify the degeneration and apoptosis patterns of photoreceptors in* rd11* mice.

## 2. Materials and Methods

### 2.1. Animals

C57BL/6J mice were purchased from the Shanghai Laboratory Animal Center (Shanghai, China), and congenic inbred strain* rd11* mice were obtained from the Jackson Laboratory (Bar Harbor, ME, USA). All mice were maintained and bred at the Wenzhou Medical University animal facilities under a 12-hour light/12-hour dark illumination cycle with an ambient light intensity of 18 lux and with free access to food and water. Wenzhou Medical University's Institutional Animal Care and Use Committee approved the animal experiments. All experiments were conducted in accordance with the Association for Research in Vision and Ophthalmology (ARVO) Statement for the Use of Animals in Ophthalmic Research. At least 5 eyes were used for each group in this study.

### 2.2. Preparation of Retinal Whole Mount and Frozen Sections

C57BL/6J and* rd11* mice at postnatal days (P) 14, 28, and 42 were euthanized by cervical dislocation. Retinal whole mount and frozen sections were prepared according to previously described methods with some modifications [23, 31–33]. Briefly, immediately following sacrifice, the eye was pressured out of the orbit and the limbus of each eye was marked at the 12 o'clock point (dorsal) using a portable cautery (Temp Cautery, AMI, ID, USA). The eyes were then enucleated and placed in 0.01 M phosphate buffered saline (PBS), and then the adipose tissue, extraocular muscles, and cornea were cut away. The eyes then were placed in fresh 4% paraformaldehyde (PFA) for 5 min, after which the lens of each eye was removed to access the eye cup, and the eyes were fixed for 20 min. The left and right eyes were noted for orientation. Retinal whole mounts were generated from each eye cup by first cutting the sclera away from the retina around the optic nerve and then gently separating the neuroretina from the eye cup. After extraction of the neuroretina, the samples were fixed for another 24 h period. Frozen sections (12-*μ*m thick) were prepared directly from eye cups, cryoprotected in 30% sucrose, and embedded in optimal cutting temperature compound (OCT).

### 2.3. Immunocytochemical Analysis with Retinal Whole Mount

Immunofluorescence staining with retinal whole mount was performed as previously described [31–34], with minor modifications. Retinal whole mounts were rinsed three times in 0.01 M PBS, for 10 min each, permeabilized in 0.3% TritonX-100 solution for 3 h, rinsed with 0.01 M PBS three times, and then blocked in 5% bovine serum albumin (BSA) for 3 h at room temperature. After that, the retinas were incubated overnight at 4°C with a rabbit polyclonal anti-human red and green (L)-cone-opsin or blue (S)-cone-opsin antibody (AB5405, AB5407; Millipore, MA, USA) that was diluted 1 : 400 in 1% BSA and 0.1% TritonX-100. After rinsing three times with 0.1% TritonX-100, the retinas were incubated for 1 h in the dark with goat anti-rabbit IgG-Cy3 (AP187C, Millipore, MA, USA) that was diluted 1 : 800 in 0.01 M PBS and then were rinsed three times with 0.1% TritonX-100 for 10 min.

The retina was cut into four quadrants, dorsal/temporal, ventral/temporal, dorsal/nasal, and ventral/nasal, at the 3, 6, 9, and 12 o'clock points under a surgical microscope. Retinal whole mount was moved gently into the PBS drop on the glass slide with 12 o'clock upward and the retinal ganglion cell layer facing upwards. PBS was suctioned into the glass slide, and the whole mount was flattened using a cover slip. If the curvature of the retina was large and could not be completely flattened, multiple cuts were made. The retinal whole mount was photographed after the addition of an antifluorescent quencher using a fluorescence microscope (Axio Imager Z1; Carl Zeiss Meditec, Oberkochen, Germany) equipped with a mercury light source and FITC or Cy3 filters. Cells labeled with L-opsin or S-opsin were manually counted in the retinal whole mount in an area that was 45 degrees of the optic nerve and at a distance of 1 mm from the optic nerve in each of the four quadrants of the retina, each within one field at 40x magnification (0.037 mm^2^). It was difficult to obtain the complete image of the retinal whole mount to account opsin density at high magnification. Here we chose the net value of the previously selected areas from 4 quadrants as the total density of each retina.

### 2.4. Immunocytochemical Analysis with Frozen Sections

Immunofluorescence staining with frozen sections was performed as previously described [23]. Frozen retinal sections were rinsed twice in 0.01 M PBS, permeabilized for 30 min in 0.3% TritonX-100, and blocked for 2 h in 5% BSA at room temperature. Then, frozen sections were incubated overnight at 4°C with a rabbit polyclonal anti-human red and green (L)-cone-opsin or blue (S)-cone-opsin antibody (AB5405, AB5407; Millipore, MA, USA) and FITC-conjugated peanut agglutinin (PNA) (B-1075, Vector Laboratories, Burlingame, CA, USA) that was diluted 1 : 400 in 1% BSA and 0.1% TritonX-100. After rinsing with 0.01 M PBS, the retinas were incubated for 1 h in a dark room with goat anti-rabbit IgG that was diluted 1 : 800 in 0.01 M PBS, followed by five rapid rinses with 0.01 M PBS. Then, the sections were incubated for 2 min in 4,6-diamidino-2-phenylindole (DAPI, dilution 1 : 500), rapidly rinsed five times with 0.01 M PBS, and photographed after the addition of antifluorescent quencher.

### 2.5. TUNEL Assay

The frozen sections from retinas were stained using in situ cell death detection kit, fluorescein (11684795910, Roche, Mannheim, Germany) according to the manufacturer's instructions. As the positive control, the retinal sections were incubated with 1000 U/ml DNase I recombinant for 10 min at room temperature to induce DNA strand breaks, prior to labeling procedures. The apoptosis was photographed using confocal laser scanning microscopy (LSM800, Carl Zeiss, Jena, Germany). The semiquantification of TUNEL staining by Image Pro plus 6.0 (Media Cybernetics, Rockville, MD, USA) was performed as described previously [35]. The ratio of integrated optical density to total area is determined as TUNEL staining quantification.

### 2.6. Gene Expression Analysis by RT-PCR

Total RNA was extracted from the frozen retinal tissues using RNeasy Mini Kit (74104, QIAGEN, Germany). RT-PCR was performed as described previously [35]; primer pairs were given in Table 1. Briefly, all the samples were run individually in RT reactions in triplicate and normalized to GAPDH. Transcripts were measured using RT-PCR, and then the data were presented with densitometric analysis as a mean of the three times in triplicate. The relative values were quantified using NIH Image J program (https://imagej.nih.gov/ij/download.html).

### 2.7. Statistical Analysis

One-way ANOVA and Dunnett's post hoc tests were performed for statistical analyses using the SPSS 18.0 Package (serial number: 10034432, SPSS Inc., Chicago, IL, USA). Five mice per group were used to analyze the differences in L-opsin and S-opsin expression in the dorsal/temporal, ventral/temporal, dorsal/nasal, and ventral/nasal quadrants of the retina and complete retina. Ten mice per group were used analyze the expression of the key genes associated with photoreceptor apoptosis. *p* values < 0.05 were considered statistically significant. Values are presented as mean ± SEM.

## 3. Results

### 3.1. Distribution of L- and S-Opsin Expression in Retinal Whole Mounts from* rd11* Mice

**Figure 1:**
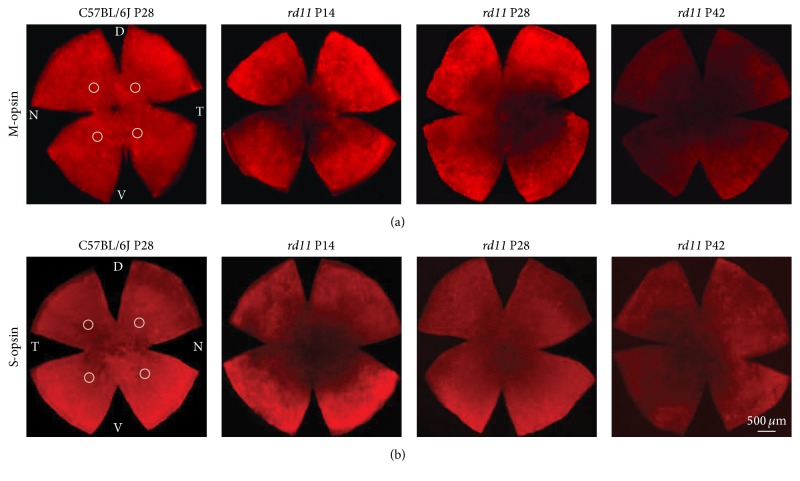
Immunofluorescence staining of L- and S-opsin in retinal whole mounts (Cy3 ×25). (a) Representative L-opsin staining in the right eye of C57BL/6J control and P14, P28, and P42* rd11* mice; (b) representative S-opsin staining in the left eye of the same C57BL/6J control and P14, P28, and P42* rd11* mice. D: dorsal; V: ventral; N: nasal; T: temporal. *n* = 5 retina per group.

In order to clarify the degeneration pattern of L-opsin and S-opsin, retinal whole mounts were stained with anti-L-opsin or S-opsin antibody. The expression of L-opsin and S-opsin did not change significantly in the dorsal/temporal, ventral/temporal, dorsal/nasal, and ventral/nasal quadrants of the wildtype C57BL/6J retina over the ages P14 to P42 (data not shown). At low magnification, similar to the C57BL/6J control mice, L-opsin expression (red fluorescence) was observed throughout the retinal whole mount in P14* rd11* mice (Figure 1(a)), and S-opsin expression (red fluorescence) was mainly observed in the ventral retina (Figure 1(b)). With increasing age, L-opsin expression gradually decreased from the center to the periphery of* rd11* retina; meanwhile, S-opsin expression gradually decreased from the dorsal/temporal to ventral/nasal quadrant of the retina (Figure 1). In P42* rd11* mice, only scattered L-opsin-positive fluorescence was detected in the dorsal/temporal quadrant of the retina, whereas scattered S-opsin-positive fluorescence was detected only in the periphery of the ventral retina (Figure 1).

**Figure 2:**
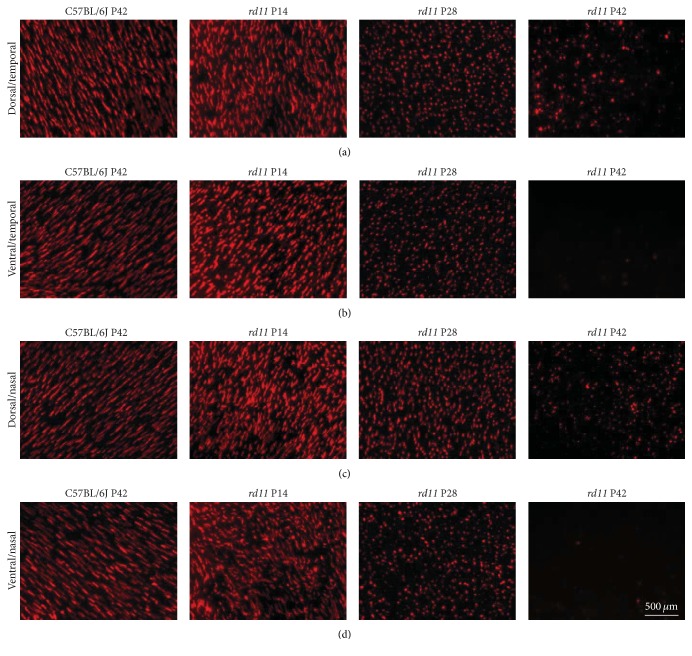
Immunofluorescence staining of L-opsin in retinal whole mounts (Cy3 ×400). (a)–(d) Representative L-opsin staining in the dorsal/temporal (a), ventral/temporal (b), dorsal/nasal (c), and ventral/nasal (d) quadrants of the retina from P42 C57BL/6J control and P14, P28, and P42* rd11* mice. *n* = 5 retina per group.

**Figure 3:**
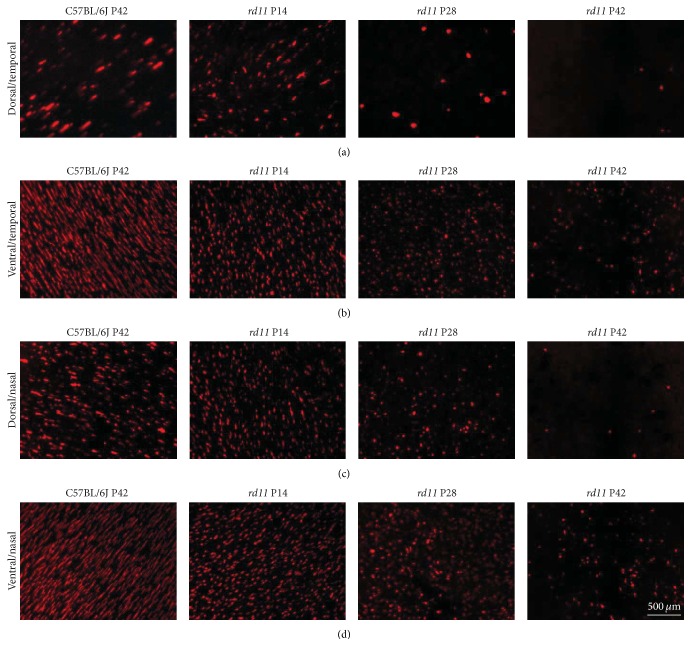
Immunofluorescence staining of S-opsin in retinal whole mounts (Cy3 ×400). (a)–(d) Representative S-opsin staining in the dorsal/temporal (a), ventral/temporal (b), dorsal/nasal (c), and ventral/nasal (d) quadrants of the retina from P42 C57BL/6J control and P14, P28, and P42* rd11* mice. *n* = 5 retina per group.

At high magnification, the degeneration patterns of L- and S-opsin were different. The typical spindle-like L-opsin-positive fluorescence was detected in most quadrants of retina in control C57BL/6J and P14* rd11* mice (Figure 2), although scattered, shorter punctate fluorescence was also detected in the ventral/nasal quadrant of the retina in wildtype and P14* rd11* mice (Figure 2(d)). With increasing age and the development of retinal degeneration, most spindle-like L-opsin-positive fluorescence changed into shorter punctate fluorescence in the P28* rd11* retina, and punctate fluorescence was significantly reduced in the P42* rd11* retina, especially in the ventral/temporal and ventral/nasal quadrants (Figure 2). In contrast to L-opsin, S-opsin was mainly detected in the ventral retina, and both punctate and fusiform fluorescence coexisted in wildtype and P14* rd11* mice (Figure 3). Compared to wildtype mice, the amount of punctuate S-opsin-positive fluorescence was significantly higher than the fusiform fluorescence in the dorsal retina of P14* rd11* mice (Figures 3(a) and 3(c)). Similar to L-opsin, all fusiform S-opsin changed into shorter punctate fluorescence in each quadrant of the retina by P28 of* rd11* mice, but only scattered punctate fluorescence remained in the ventral retina by P42 of* rd11* mice (Figure 3(d)).

**Figure 4:**
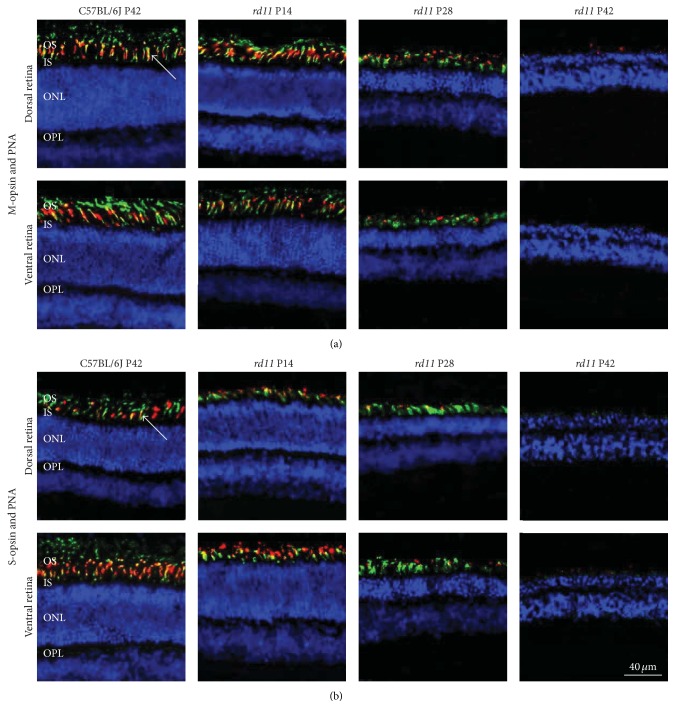
Costaining for L-opsin or S-opsin and PNA in C57BL/6J control and* rd11* retina. Immunofluorescence of retinal cryosections from C57BL/6J control and P14, P28, and P42* rd11* mice labeled with L-opsin or S-opsin and PNA lectin. (a) Costaining of L-opsin and PNA. (b) Costaining of S-opsin and PNA. Cells were counterstained with DAPI. Arrows indicate cones with colocalization of PNA and either L- or S-opsin. Red: L-opsin or S-opsin, green: PNA, and blue: DPAI. OS: outer segments; IS: inner segments; ONL: outer nuclear layer; OPL: outer plexiform layer. *n* = 5 retina per group.

To determine the degeneration patterns of L-opsin and S-opsin in* rd11* mice, PNA, the cone-specific marker, which specifically binds to the cone interphotoreceptor matrix sheath [36], was used to identify the overall cone cells combined with L- or S-opsin staining to identify specific L- or S-cone cells with frozen section. In wildtype and* rd11* mice, we detected coexpression of PNA and L-opsin/S-opsin (Figure 4). In wildtype mice, both L- and S-opsins were detected in the outer segments of the cones (Figure 4). Abundant L-opsin was also detected in P14* rd11* mice; however, the S-opsin-positive outer segments became shorter in the dorsal retina, which matched the punctuate S-opsin-positive fluorescence in the retinal whole mount. In the P28* rd11* retina, we detected less S-opsin- and L-opsin-positive fluorescence and shorter cone outer segments; few L/S-opsin- and PNA-positive cones were detected in P42* rd11* mice (Figure 4). Similar to the expression pattern of the retinal whole mounts, L-opsin expression in the dorsal retina was higher than that in the ventral retina (Figure 4(a)), whereas S-opsin expression in the ventral retina was higher than that in the dorsal retina in retinal cryosections (Figure 4(b)). Our data suggest that most cone-opsins and structures have been lost by P42 in* rd11* mice (Figures 2–4).

### 3.2. Comparison of L- and S-Opsin Density in the* rd11* Retina at Different Ages

There was no significant difference in the L-opsin or the S-opsin expression density in the dorsal/temporal, ventral/temporal, dorsal/nasal, and ventral/nasal quadrants of the retina or complete retina in P14* rd11* mice when compared to expression density in the age-matched control C57BL/6J retina (*p* > 0.05). In the P28 and P42* rd11* retinas, there was a significant decrease in both L-opsin and S-opsin expression densities in the four quadrants and complete retina when compared to the expression densities in the age-matched control retinas (L-opsin and S-opsin in the four quadrants and complete retina, *p* < 0.001, except P28: L-opsin in dorsal/temporal quadrant, *p* < 0.01) (Tables 2 and 3).

There were no significant differences in the L-opsin and S-opsin density in the four quadrants and complete retina of the control C57BL/6J mice at all ages examined (P28 versus P14, P42 versus P28, and P42 versus P14), except for a significant decrease in S-opsin in the dorsal/temporal quadrant of the P28 or P42 retina compared with the P14 retina. With increasing age, the expression of L-opsin and S-opsin decreased significantly in the four quadrants and complete retina of the* rd11* mice (*p* < 0.01, P28 versus P14, P42 versus P28, and P42 versus P14), except for L-opsin in the dorsal/temporal quadrant of the P28 retina compared with the P14 retina (*p* > 0.05) and S-opsin in the dorsal/temporal quadrant of the P42 retina compared to the P28 retina (*p* > 0.05) (Tables 2 and 3).

### 3.3. Apoptosis Pattern of Photoreceptor Cells in* rd11* Mice

**Figure 5:**
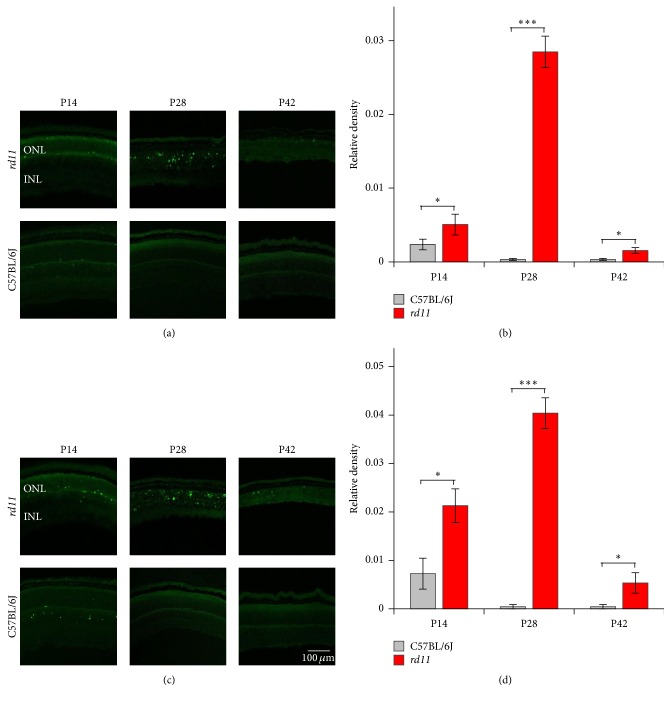
TUNEL staining in C57BL/6J control and* rd11* retina. (a) and (c) Central (a) and peripheral (c) retina from P14, P28, and P42 C57BL/6J and* rd11* mice. (b) and (d) The quantification of positive signals from (a) and (c). ONL: outer nuclear layer; INL: inner nuclear layer. ^*∗*^*p* < 0.05; ^*∗∗∗*^*P* < 0.001. *n* = 5 retina per group.

Apoptosis is a common feature of photoreceptor degeneration [28]; we analyzed the change of apoptosis in the retina by TUNEL staining. In P14* rd11* mice, the structure of retina remained substantially intact, and few apoptotic-positive signals were detected in the outer nuclear layer of central and peripheral retina. In P28* rd11* mice, apoptotic-positive signals were significantly increased in the central and peripheral retina. In P42* rd11* mice, the apoptotic-positive signals were only sporadically detected (Figure 5). In wildtype mice, few apoptotic-positive signals were only detected in P14 retina. We did not find apoptotic-positive signals in P28 and P42 wildtype retina (Figure 5).

**Figure 6:**
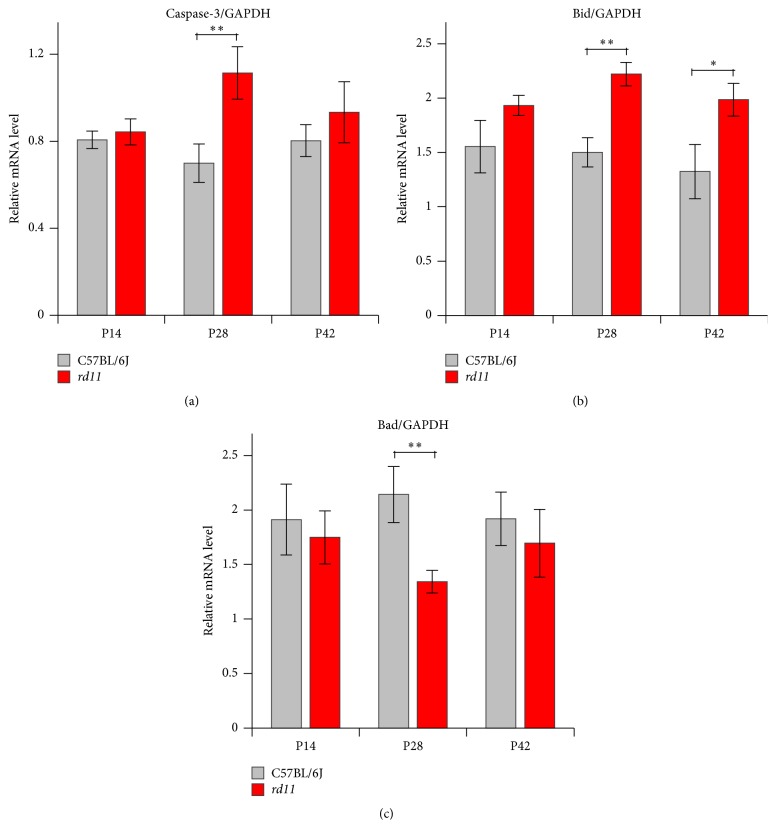
Gene expression related to apoptotic signal pathway. (a)–(c) Gene expression of Caspase-3 (a), Bid (b), and Bad (c) at mRNA levels by RT-PCR in P14, P28, and P42 C57BL/6J and* rd11* retina. ^*∗*^*p* < 0.05; ^*∗∗*^*p* < 0.01. *n* = 10 retina per group.

We also analyzed mRNA expression of the key genes associated with photoreceptor apoptosis. Compared to wildtype mice, the expressions of proapoptotic factors Caspase-3 and Bid were significantly upregulated (Figures 6(a) and 6(b)); the expression of antiapoptotic factor Bad was significantly downregulated in the P28* rd11* retina (Figure 6(c)). These alterations are consistent with the results of TUNEL staining (Figure 5). In the P42* rd11* retina, the level of Bid was significantly upregulated compared to P42 wildtype mice (Figure 6(b)). Compared to P14* rd11* retina, the expressions of Caspase-3 and Bid were significantly upregulated; while the expression of Bad was significantly downregulated in the P28* rd11* retina (Figure 6).

## 4. Discussion

In the rodent retina, the cell type, cell number, and distribution pattern of the rod and cone photoreceptors define the specific function of the visual system [19]. The distribution of cone photoreceptors differs in different species [37]. Short wavelength and middle-to-long-wavelength-sensitive cone photoreceptors have different functions [38]. It is crucial to clarify the distribution and degeneration pattern of L-opsin and S-opsin in* rd11* mice. When compared to previous studies [19–21], our study demonstrated in detail the slight change of L-opsin and S-opsin expression. We found that the expression of L-opsin gradually decreased from the dorsal/temporal to ventral/nasal quadrant, whereas the expression of S-opsin gradually decreased from the ventral/nasal to dorsal/temporal quadrant and decreased drastically in the periphery of dorsal/temporal quadrant in C57BL/6J mice. Likewise, we found that most of the L- or S-opsin-positive fluorescence was fusiform in the C57BL/6J mice (Figures 2 and 3), but only L- and S-opsin-positive punctate fluorescence was found from P28 and older* rd11* mice. The shortened opsin-positive punctate fluorescence may be due to shortened outer segments and reduced opsin expression in aged* rd11* mice. In fact, S-opsin-positive punctate fluorescence was detected in the P14* rd11* retinal whole mount, although there were no significant differences for the L- or S-opsin density in the P14* rd11* entire retina compared to the control retina, which suggested that cone photoreceptors began to degenerate at P14 of* rd11* mice. These data suggest that the degeneration of short wavelength-sensitive cone photoreceptors occurred earlier, which is similar to the degeneration seen in* rd12* mice that carry the RPE65 mutation [27, 39].

Similar to other mouse models with cone photoreceptors degeneration (*Cpfl5* and* rd12* mice) [40, 41], our data suggest that L-opsin degeneration initiated around the optic nerve and spread to the peripheral retina, from the ventral and nasal sides and then to the dorsal and temporal sides of the* rd11* retina. S-opsin degeneration spread from the temporal and dorsal sides to the nasal and ventral sides of the retina with increasing age in* rd11* mice, which was consistent with the general rule of S-opsin change in* rd12* mice [41]. Although some ligands regulators or transcription factors, such as retinoic acid, thyroid hormone, Delta, and Notch, can regulate the expression of L-opsin and S-opsin [19, 42–45], the specific mechanism of regional differences and gradient change in L-opsin and S-opsin degeneration of* rd11* mice remained unclear. Our data showed that the first degenerating S-opsins were mainly located at ventral retina, which might be the least necessary function for seeing blue sky in mice; and the latest degenerating L-opsins were around the dorsal retina, which should be the most useful function for mice. Those observations may suggest that the cone degeneration pattern may be related to evolution. In the P42* rd11* retina, the expression of L-opsin, S-opsin, and PNA was very weak and almost disappeared in some quadrants, which suggests that the degeneration of cone photoreceptors was very advanced by P42. The degeneration patterns of L- and S-opsin were consistent with changes in retinal electrophysiology and histopathology in* rd11* mice [10].

Because of field of vision limitations under high magnification, we only calculated the expression density of L-opsin and S-opsin in a small area of the retina (0.037 mm^2^). Since the cone degeneration was not uniform in the* rd11* retina, this study only focused on the general pattern of L- and S- opsin degeneration. In fact, we noted that there were slight differences in the expression of L-opsin and S-opsin at the distance of 1 mm and 2 mm from the optic disc in the* rd11* retina. Although there was no significant difference in the expression of S-opsin in the dorsal/nasal and ventral/nasal quadrants near the optic nerve, S-opsin expression in the dorsal/nasal quadrant was closer to the optic nerve in the P14* rd11* retina, so the field of view was very important to the analysis of the final results.

As early as 1981, scholars had suggested immunofluorescence staining with retinal whole mount as a method for observing retinal ganglion and photoreceptor cells [46–48]. With the development of research and technology, immunofluorescence staining with retinal whole mount has been widely used and improved [26, 49, 50]. In this study, we continued to improve this technique, resulting in a better procedure that was easy to operate and that was suitable for batch specimens. In contrast to the previous studies, we paid extra attention to the following points during the process. (1) It was critical to select the appropriate fixation time, which determined the qualities of the retinal whole mounts. A minimal 5-min fixation of the eyes in 4% PFA prior to removing the lens was necessary to prevent the collapse of the eye cups. (2) Sufficient permeabilization was a key factor that affected the staining results; therefore, the retinal whole mounts and frozen sections needed to be soaked sufficiently in TritonX-100.

Similar to the degeneration pattern of photoreceptor cells, we found that apoptosis began at P14 of* rd11* mice, and the apoptotic signal was strongest at P28 of* rd11* mice. Because most photoreceptor cells had been lost, the apoptotic signal was very weak at P42 of* rd11* mice. Our data showed that the P14 of* rd11* mice was a key point for gene therapy.

In 2008, American and British scientists proved that gene therapy could restore most of the visual function at middle to late stages of LCA2 patients in phase I clinical trial [51, 52]. In LCA2 clinical trial, because the patients were at middle to late stages of diseases, the photoreceptor cells had begun irreversible degeneration or apoptosis before treatment. So the loss of photoreceptor cells continued after treatment, and the long-term efficacy was not optimistic. In order to solve the above problem and better simulate human clinical trials, we are conducting an improved experiment to treat the advanced diseases, which is based on gene replacement therapy combined with antiapoptotic agents, histone deacetylase inhibitors. Our purpose is to maintain a long-term visual function after gene therapy in* rd11* mice.

Therefore, it is very important for the long-term efficacy of gene therapy to define the start time of the photoreceptor degeneration. The treatment before photoreceptor degeneration can achieve long-term efficacy. We analyzed the degeneration and apoptosis pattern of photoreceptor cells and defined the start time of the degeneration in earlier stage of* rd11* mice. Our studies were very helpful to our ongoing improved trial for gene therapy in* rd11* mice.

## 5. Conclusion

In our study, we analyzed the distribution and degeneration pattern of L- and S-opsin in different ages of* rd11* retinas using immunofluorescence staining with retinal whole mount and frozen sections. We found that L-opsin and S-opsin had a different distribution and degeneration pattern in* rd11* mice. The degeneration or apoptosis of photoreceptor cells began at P14 of* rd11* mice. With an increase in age, the expression of L-opsin and S-opsin decreased significantly in* rd11* mice. Most of the L-opsin and S-opsin degenerated in six weeks after birth in* rd11* mice. Determining the different degeneration patterns or apoptotic process may benefit the treatment strategy of similar retinal degenerative diseases. Based on these findings, a gene replacement therapy combined with antiapoptosis therapy using* rd11* mice is ongoing.

## Figures and Tables

**Table 1:** Primer pairs for RT-PCR.

Gene	Primer pairs	Product size (bp)
Caspase-3	F: 5′-AGCTTCTTCAGAGGCGACTA-3′	381
	R: 5′-GGACACAATACACGGGATCT-3′	
Bad	F: 5′-GAGGAAGTCCGATCCCGGAA-3′	494
	R: 5′-CGGCGCTTTGTCGCATCTGT-3′	
Bid	F: 5′-CCTGCTGGTGTTCGGCTTTC-3′	466
	R: 5′-CGTGTGGAAGACATCACGGA-3′	
GAPDH	F: 5′-CCCATGTTTGTGATGGGTGT-3′	134
	F: 5′-CCTTCCACAATGCCAAAGTT-3′	

**Table 2:** Comparison of L-opsin density in retinal whole mounts of C57BL/6J control and *rd11* mice.

Location	P14		P28		P42	
	C57BL/6J	*rd11*	C57BL/6J	*rd11*	C57BL/6J	*rd11*
DT	490 ± 14	473 ± 15	486 ± 11	410 ± 14^a^	430 ± 9	157 ± 10^b,c^
VT	404 ± 5	415 ± 8	444 ± 10	292 ± 4^b,c^	420 ± 17	2 ± 1^b,c^
DN	445 ± 7	463 ± 10	467 ± 17	316 ± 8^b,c^	471 ± 6	83 ± 7^b,c^
VN	393 ± 7	370 ± 11	442 ± 7	243 ± 11^b,c^	407 ± 12	2 ± 1^b,c^
M/T	1730 ± 34	1717 ± 33	1819 ± 71	1281 ± 43^b,c^	1740 ± 52	256 ± 20^b,c^

Different letter superscripts (a, b, and c) are significantly different. Values are presented as mean ± SEM. ^a^*p* < 0.01 and ^b^*p* < 0.001, *rd11* retina versus age-matched control retina; ^c^*p* < 0.001, the comparison of *rd11* retina at indicated ages. DT: dorsal/temporal; VT: ventral/temporal; DN: dorsal/nasal; and VN: ventral/nasal quadrants of retina. M/T: mean of total density in the complete retina. *n* = 5 retina per group.

**Table 3:** Comparison of S-opsin density in retinal whole mounts of C57BL/6J control and *rd11* mice.

Location	P14		P28		P42	
	C57BL/6J	*rd11*	C57BL/6J	*rd11*	C57BL/6J	*rd11*
DT	180 ± 11	172 ± 10	69 ± 7	9 ± 2^a,c^	71 ± 6	2 ± 1^a^
VT	395 ± 14	382 ± 9	427 ± 14	164 ± 9^a,c^	426 ± 18	40 ± 8^a,c^
DN	363 ± 9	340 ± 12	383 ± 13	68 ± 4^a,c^	382 ± 14	4 ± 1^a,c^
VN	432 ± 15	451 ± 17	474 ± 9	310 ± 8^a,b^	469 ± 14	107 ± 15^a,c^
M/T	1339 ± 45	1360 ± 52	1384 ± 40	562 ± 19^a,c^	1362 ± 50	156 ± 21^a,c^

Different letter superscripts (a, b, and c) are statistically different. Values are presented as mean ± SEM. ^a^*p* < 0.001, *rd11* retina versus age-matched control retina; ^b^*p* < 0.01 and ^c^*p* < 0.001, the comparison of the *rd11* retina at indicated ages. DT: dorsal/temporal; VT: ventral/temporal; DN: dorsal/nasal; and VN: ventral/nasal quadrants of retina. M/T: mean of total density in the complete retina. *n* = 5 retina per group.
